# Cor Triatriatum Dexter With a Sinus Venosus Atrial Septal Defect in a 50-Year-Old Woman: A Case Report

**DOI:** 10.7759/cureus.53477

**Published:** 2024-02-02

**Authors:** Hasan Kazma, Malak Fakih, Ali Raad, Aalaa Saleh, Malek Mohammed

**Affiliations:** 1 Cardiology, Bahman Hospital, Beirut, LBN; 2 Medicine, Lebanese University Faculty of Medical Sciences, Beirut, LBN; 3 Cardiology, Lebanese University Faculty of Medicine, Beirut, LBN; 4 Pulmonary and Critical Care, Bahman Hospital, Beirut, LBN; 5 General Medicine, Lebanese University Faculty of Medical Sciences, Beirut, LBN; 6 Invasive Cardiac Laboratory, Bahman Hospital, Beirut, LBN

**Keywords:** right cardiac chambers dilatation, pulmonary hypertension, pulmonary to systemic flow ratio, cor triatriatum dexter, anomalous venous return, sinus venosus atrial septal defect, functional tricuspid valve regurgitation, atrial septal defects, adult congenital heart disease, transthoracic and transesophageal echocardiography

## Abstract

The diagnosis of atrial septal defect (ASD) may be delayed until adulthood or even later in life as it is a well-tolerated congenital heart disease. If patients are not examined and investigated well in childhood, the diagnosis may be delayed until later in adulthood when patients present with palpitations and sometimes dyspnea due to the right chambers dilatation from right ventricular volume overload. In this report, we present a case of a 50-year-old female patient with symptoms of heart failure and atrial fibrillation who was found to have dilated right cardiac chambers, dilated pulmonary artery, severe tricuspid regurgitation, pulmonary hypertension, and a pulmonary-to-systemic flow ratio (Qp/Qs) of more than 1.5 by transthoracic echocardiography and Doppler, indicating left to right shunt at the atrial level. However, transthoracic echocardiography could not visualize the defect, and two-dimensional (2D) transesophageal echocardiography was done in this patient and documented the presence of a sinus venosus ASD with an incomplete cor triatriatum dexter membrane; all four pulmonary veins were identified going to the left atrium. Since the presence of an incomplete cor triatriatum dexter membrane (despite causing no symptoms) makes the percutaneous closure of the sinus venosus ASD and the percutaneous repair of tricuspid regurgitation very difficult, we decided to advise surgical ASD closure and tricuspid valve repair for the patient.

## Introduction

Adult patients presenting with symptoms of palpitations, fatigue, and or dyspnea, in addition to having right cardiac chambers dilatation, must be investigated thoroughly to rule out atrial septal defect (ASD). In fact, four types of ASDs exist: (i) ostium primum and (ii) ostium secundum ASDs, which are the anatomical defects involving the interatrial septum, and (iii) sinus venosus ASDs and (iv) the unroofed coronary sinus, which exhibit physiological behaviors similar to traditional interatrial septal defects. While 65-75% of patients with osteum secundum ASD are females, the gender distribution is equal for the other types [1].

Sinus venosus ASD is usually difficult to visualize using transthoracic echocardiography (TTE). Suggestive signs of any type of ASD are dilated right cardiac chambers, a dilated pulmonary artery, an elevated early E tricuspid velocity, tricuspid regurgitation, pulmonary hypertension, and a pulmonary to systemic flow ratio (Qp/Qs) of more than 1.5 indicating left to right shunt [2]. Two-dimensional (2D) and four-dimensional (4D) transesophageal echocardiography (TEE) are the gold standard for the visualization of ASD including the sinus venosus ASD and can help in the diagnosis of associated partial anomalous pulmonary venous return (PAPVR) to the right atrium if present [3-5].

Other cardiac imaging modalities including cardiac CT angiogram (CTA) and cardiac magnetic resonance imaging (MRI) are very important and powerful tools for the diagnosis of ASDs, and associated anomalies like a PAPVR to the right atrium, superior vena cava (SVC), or inferior vena cava (IVC). These imaging procedures should be done before contemplating percutaneous closure of ASD because they can assess the defect and adjacent structure more readily to decide on the feasibility of the percutaneous closure, but more importantly, they can demonstrate more precisely the PAPVR. When these anomalies are present in patients with ASD, surgical correction is preferred over percutaneous repair because percutaneous treatment of ASD (including sinus venosus type) with PAPVR is very difficult and limited to the pulmonary veins draining to the right atrium [5-7].

The presence of an incomplete cor triatriatum dexter membrane is usually asymptomatic as it is a bystander; it has a different embryogenic origin from that of the atrial septum. The cor triatriatum dexter membrane can be identified by TTE, TEE, cardiac CTA, or cardiac MRI [8,9]. The association of incomplete cor triatriatum dexter membrane and a sinus venosus ASD is very rare, and makes the percutaneous closure of ASD [8-10] and the percutaneous treatment of tricuspid valve regurgitation [11,12] very challenging.

Our patient was misdiagnosed initially as having chronic bronchitis with secondary right cardiac chambers dilatation, severe tricuspid regurgitation, and pulmonary hypertension (cor-pulmonal) in another facility. It was our documentation by 2D TTE and Doppler of the presence of pulmonary to systemic flow ratio (Qp/Qs) of more than 1.5 that allowed us to suspect the presence of left to right shunt at the atrial level [2]. We could not, however, visualize the ASD defect by TTE, and TEE helped visualize a sinus venosus defect and an incomplete cor triatriatum dexter membrane. The patient was advised surgical repair for both sinus venosus ASD and tricuspid regurgitation.

## Case presentation

A 50-year-old female patient was admitted to our hospital with a five-day history of fever, cough, and sputum production associated with dyspnea, orthopnea, and palpitations.

**Figure 1:**
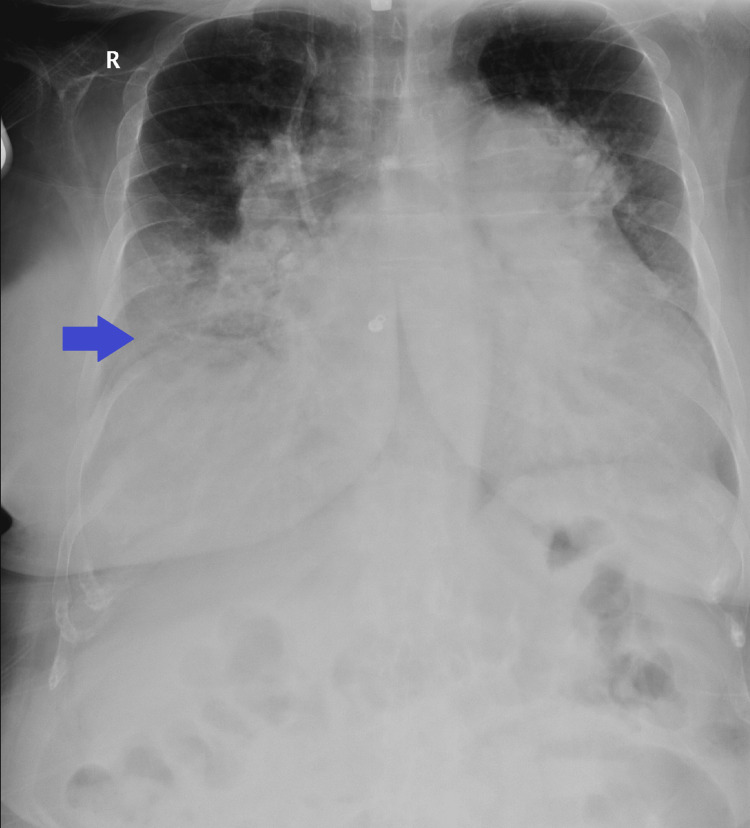
Chest X-ray showing cardiomegaly and right lower lobe infiltrates (blue arrow).

The patient had a history of a gunshot wound to the head at the age of 21 years with residual right-sided neurologic hemiparesis. She was a heavy smoker (one pack daily for 30 years) with the diagnosis of chronic bronchitis, heart failure (cor pulmonal), and persistent atrial fibrillation diagnosed in another institution, where she was started on ramipril 5 mg orally daily, metoprolol 50 mg orally daily, and apixaban 5 mg orally twice daily. On physical examination, she had tachypnea with a respiratory rate of 20 per minute, temperature of 38.5 degrees Celsius, and blood pressure of 120/80. The heart rate was irregular at 90 beats per minute and oxygen saturation was maintained at 94% by finger pulse oximetry while the patient was breathing room air. On physical exam, she had mild neck vein distension, a systolic murmur over the right sternal border on cardiac auscultation, and diffuse wheezing and rhonchi on lung examination. The abdomen was soft with no hepatomegaly and no splenomegaly. Mild leg edema was noted bilaterally. All pulses were adequate. ECG showed atrial fibrillation with a ventricular response at 96 beats per minute and an incomplete right bundle branch block. Chest x-ray showed cardiomegaly (Figure 1) with right lower lobe infiltrates. Complete blood count and differential showed a WBC at 15,000 cell/µl with 85% segmented and 12% lymphocytes, C-reactive protein was elevated at 190 mg/L, the blood urea nitrogen, creatinine, electrolytes, calcium, magnesium, and complete liver enzymes including albumin and prothrombin time were normal. A thyroid stimulating hormone (TSH) test was done and it was normal. Due to the concern of right lower lobe pneumonia, she was started on intravenous antibiotics after blood and urine cultures: ceftriaxone 2 g daily was started intravenously, inhaled bronchodilators salbutamol 0.3 mg every six hours, and ipratropium bromide 500 micrograms every six hours by nebulizer were also started. The patient was kept on metoprolol 50 mg once daily orally, ramipril 5 mg once daily orally, and apixaban 5 mg twice daily orally. Furosemide was started at the dose of 20 mg daily orally. The patient's clinical condition improved within 24 hours.

**Figure 2:**
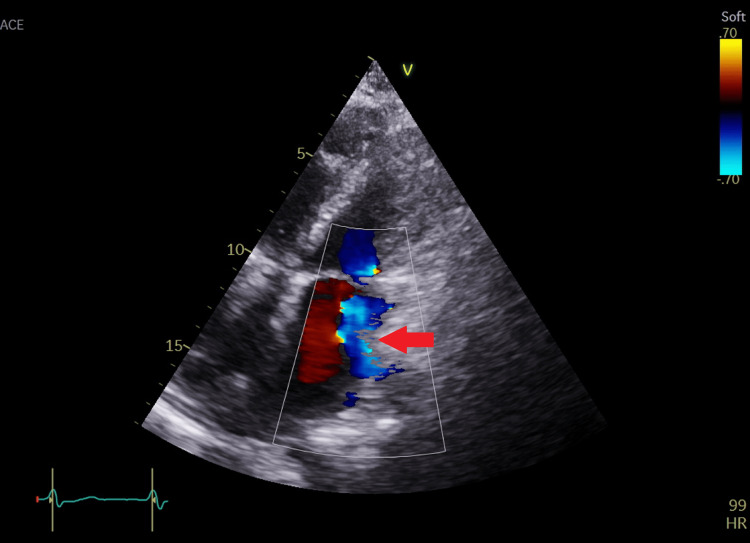
Apical four-chamber view with color coding of the mitral valve showing mild mitral regurgitation.

**Figure 3:**
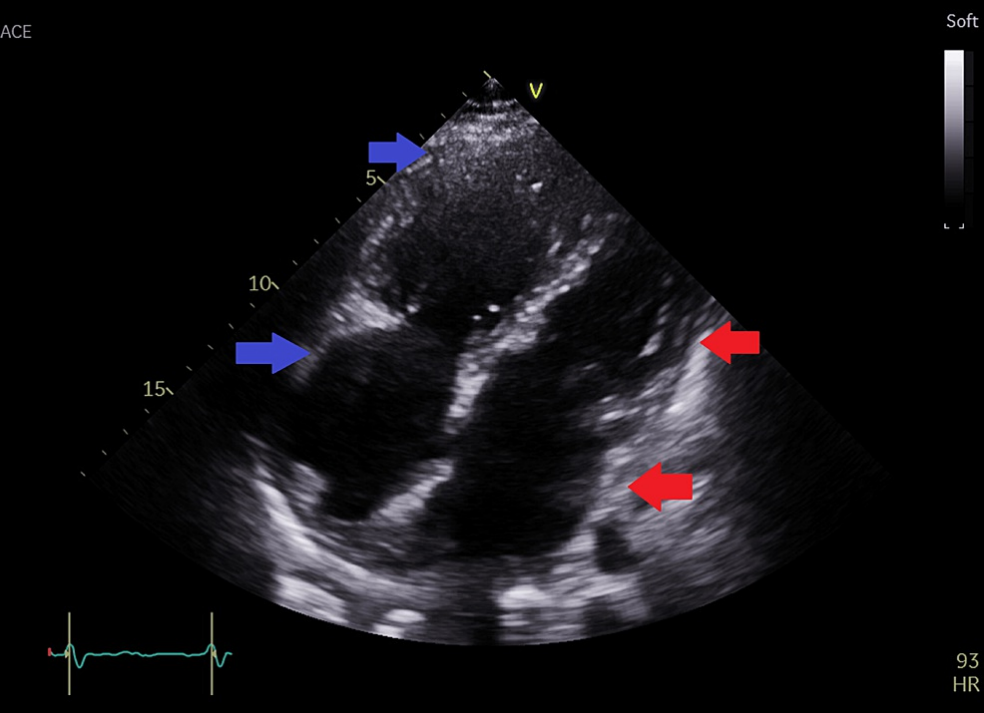
Modified apical four-chamber view showing dilated right atrium and right ventricle. blue arrows=right atrium and right ventricle; red arrows=left atrium and left ventricle

**Figure 4:**
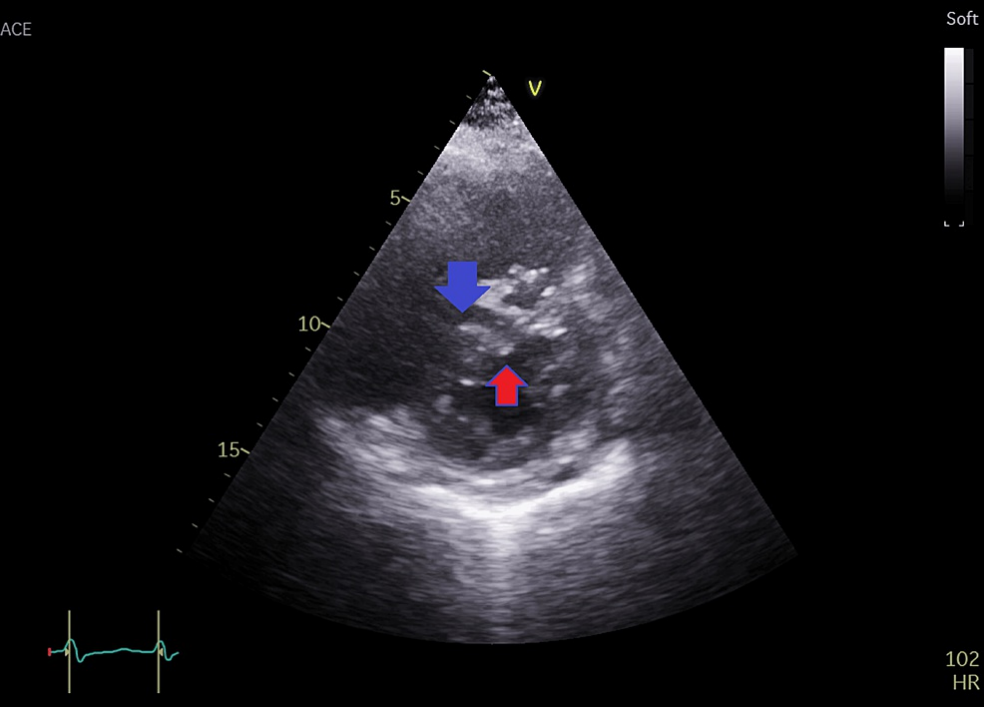
Left parasternal short-axis view of the left and right ventricles showing dilated right ventricle (blue arrow) with right ventricular volume overload: D-shaped left ventricle (red arrow) in diastole

**Figure 5:**
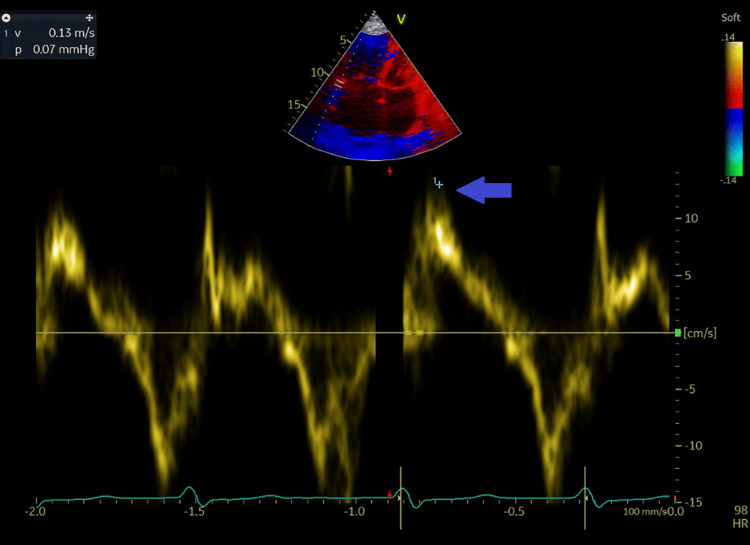
Tricuspid annulus velocity at lateral level by tissue pulsed wave Doppler from the apical window showing normal right ventricular systolic function with an S' at 13 cm/second (blue arrow).

**Figure 6:**
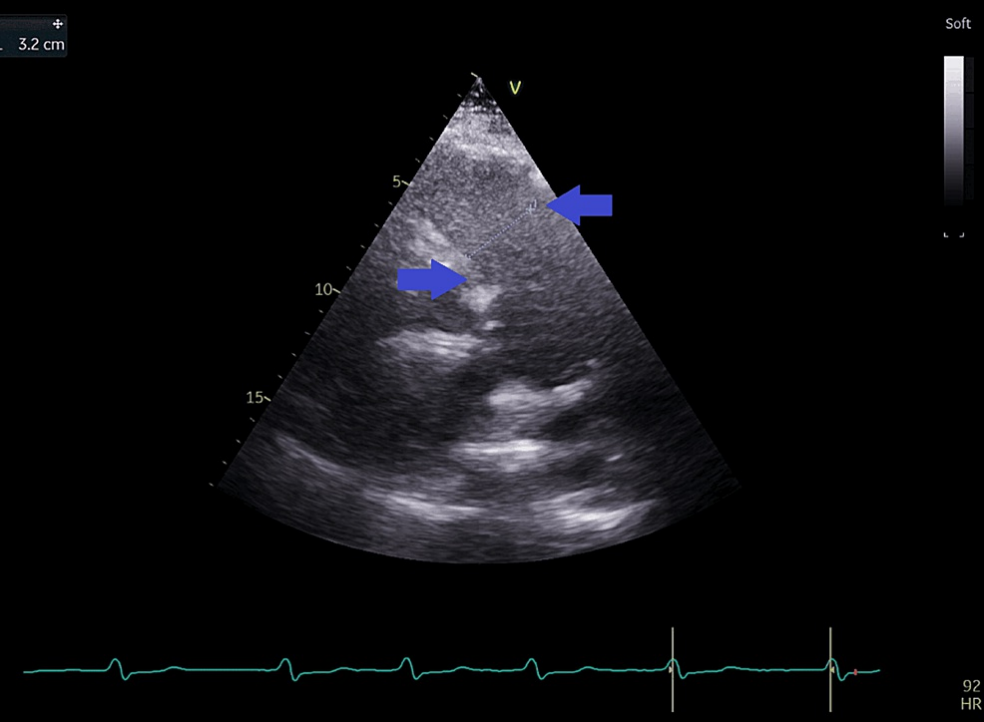
Left parasternal short-axis view at the base of the heart showing a moderately dilated pulmonary artery (blue arrows).

**Figure 7:**
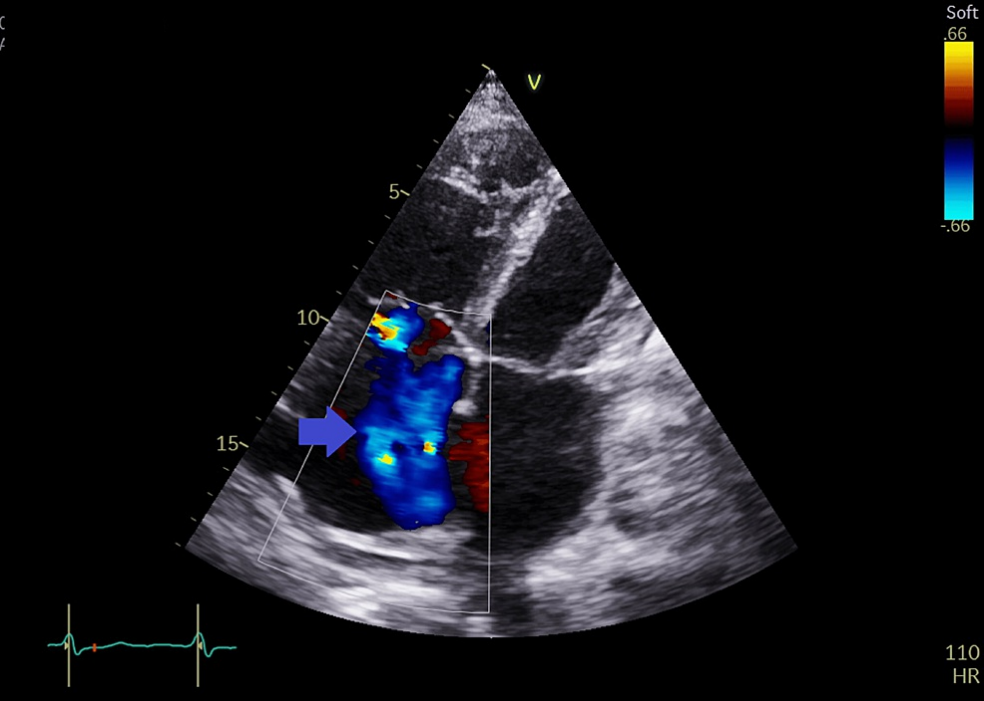
Apical four-chambers view with color coding of the tricuspid valve showing severe tricuspid regurgitation (blue arrow).

**Figure 8:**
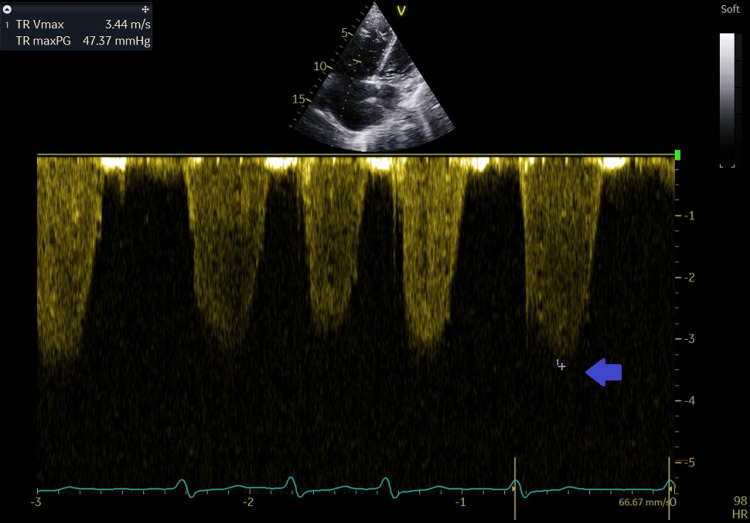
Tricuspid regurgitation jet velocity recorded with continuous wave Doppler at 3.4 m/second (blue arrow).

**Figure 9:**
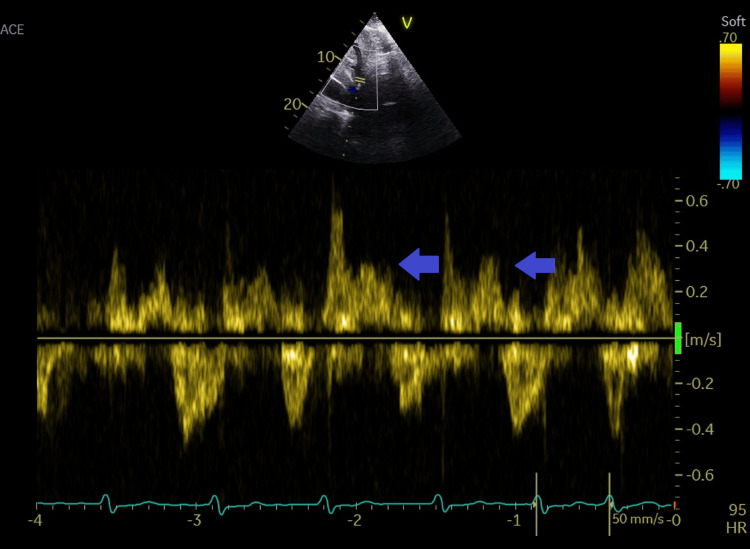
Pulsed wave Doppler recording in the hepatic vein showing systolic reversal of flow, indicating severe tricuspid regurgitation. Blue arrows show systolic reversal spectral display

**Figure 10:**
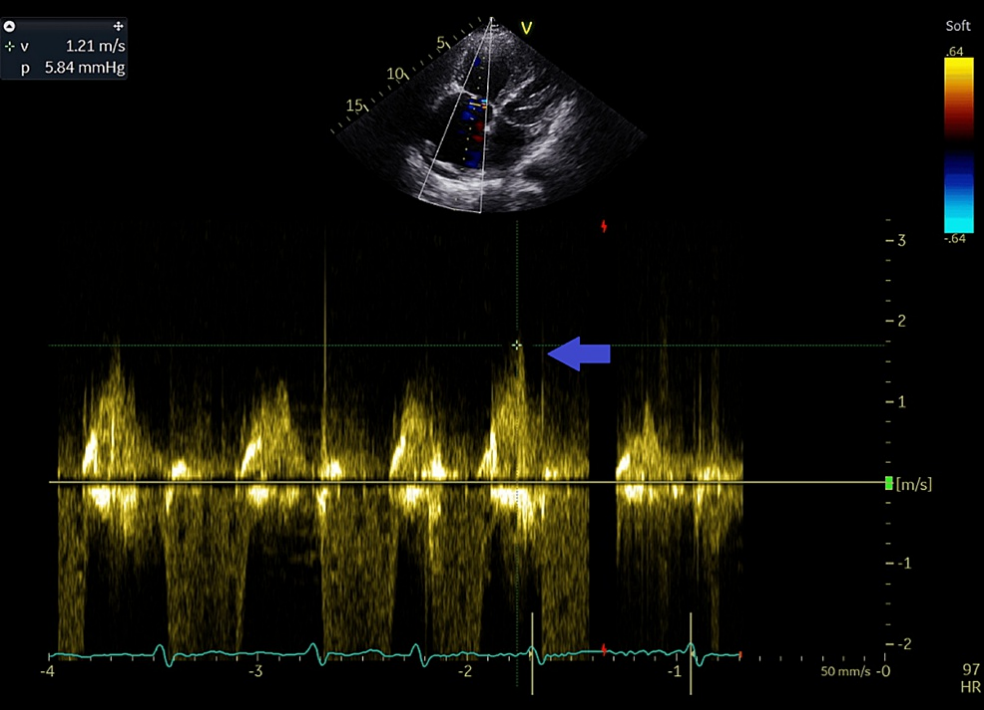
Tricuspid E velocity by continuous wave Doppler recorded at 1.2 m/second from apical four-chamber view. Blue arrow shows the E velocity spectral display

THE showed a mildly dilated left atrium, normal left ventricular size and function, mild mitral regurgitation (Figure 2), moderately dilated right atrium and right ventricle (Figure 3) with evidence of volume overload of the right ventricle, D-shaped left ventricle in diastole (Figure 4), normal right ventricular systolic function as documented by an S' tricuspid systolic annular velocity at lateral level with tissue Doppler at 13 centimeters per second (Figure 5), moderately dilated pulmonary artery (Figure 6), and severe tricuspid regurgitation (Figure 7) allowing estimation of systolic pulmonary artery pressure at 52 mmHg. Tricuspid regurgitation velocity was at 3.4 meters per second by continuous wave (CW) Doppler (Figure 8). The severity of the tricuspid regurgitation was further documented by the systolic reversal of flow in the hepatic veins by pulsed wave (PW) Doppler (Figure 9). The tricuspid E velocity by CW Doppler was increased at 1.2 meters per second (Figure 10).

**Figure 11:**
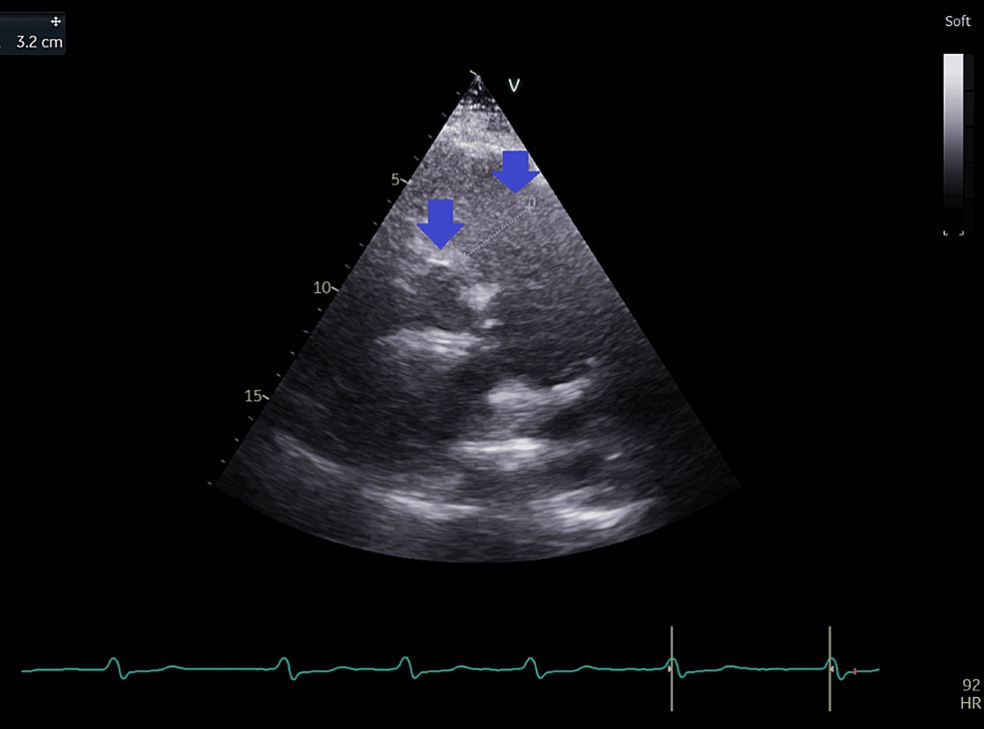
Short-axis view at the base of the heart from the left parasternal window with measurement of the right ventricular outflow tract diameter at 3.2 cm (blue arrows).

**Figure 12:**
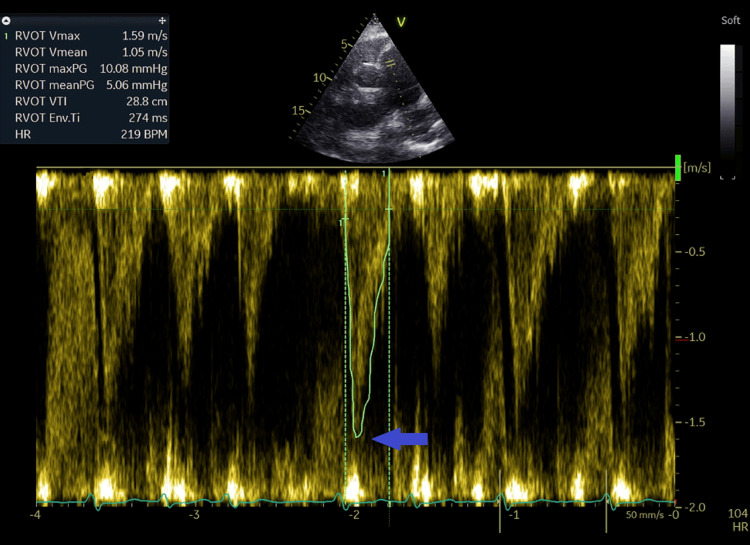
Short-axis view at the base of the heart from the left parasternal window with measurement of right ventricular outflow tract velocity at 1.59 m/second and time velocity integral at 28.8 cm (blue arrow).

**Figure 13:**
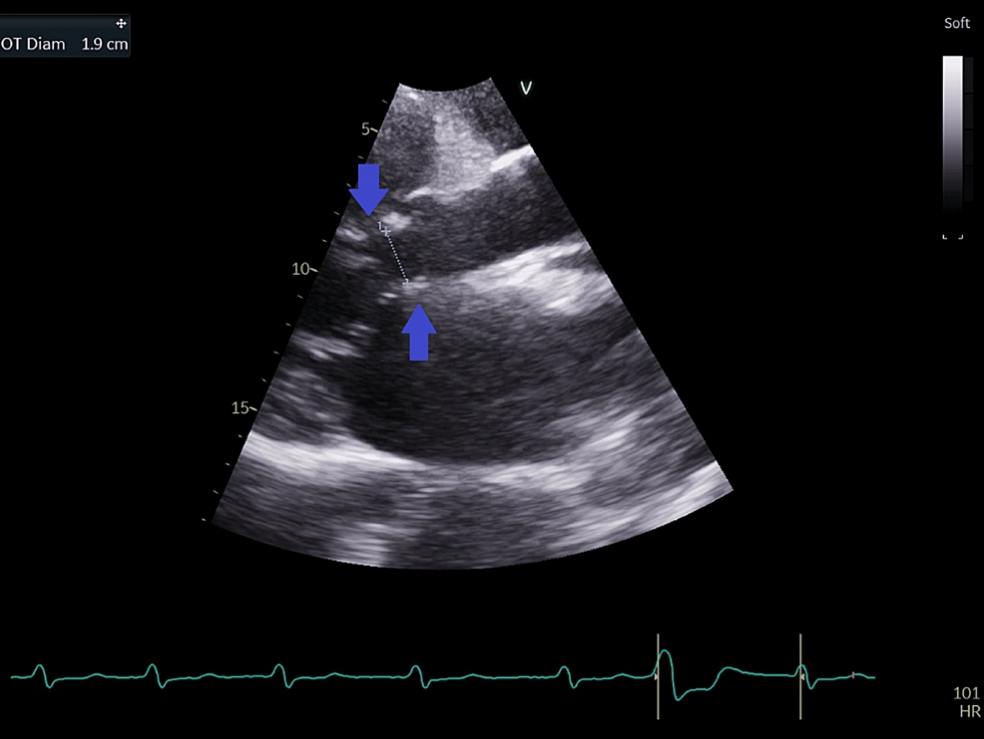
Parasternal long-axis view with measurement of the left ventricular outflow tract diameter at 1.9 cm (blue arrow).

**Figure 14:**
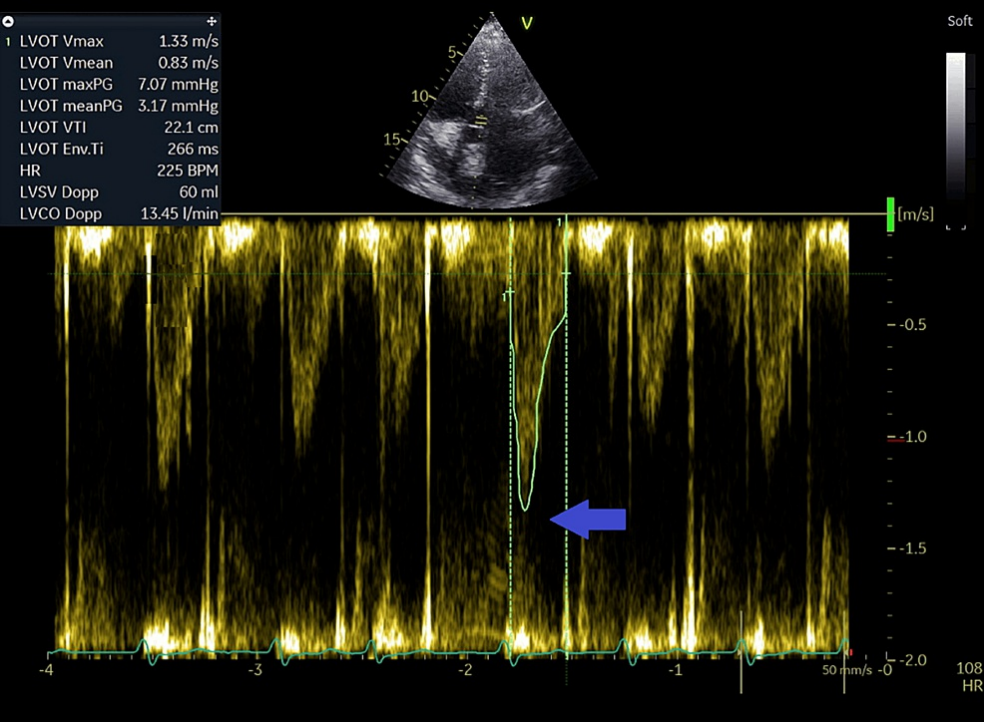
Measurement of the left ventricular outflow tract velocity at 1.3 m/seconds and time velocity integral at 21.1 cm from the apical five-chamber view (blue arrow).

Due to the concern of left to right shunt at the atrial level, the pulmonary to systemic flow (output) ratio (Qp/Qs) was calculated; the Qp/Qs shunt was 3.7: right ventricular outflow tract (RVOT) diameter=3.2 cm (Figure 11), RVOT time velocity integral (RVOT TVI)=28.8 cm (Figure 12): Qp=(π÷4) x (RVOT diameter x RVOT diameter) x RVOT TVI= 0.785 x (3.2x3.2) x 28.8=231.5 ml; Left ventricular outflow diameter (LVOT)=1.9cm (figure 13), LVOT TVI=22.1cm (figure 14): Qs=(π÷4) x (LVOT diameter x LVOT diameter) x LVOT VTI= 0.785 x (1.9x1.9) x 22.1= 62.6 ml (Qp/Qs=231.5/62.6=3.7). This elevated Qp/Qs at 3.7 indicated a left-to-right shunt at the atrial level. Thus, a TEE was done.

**Figure 15:**
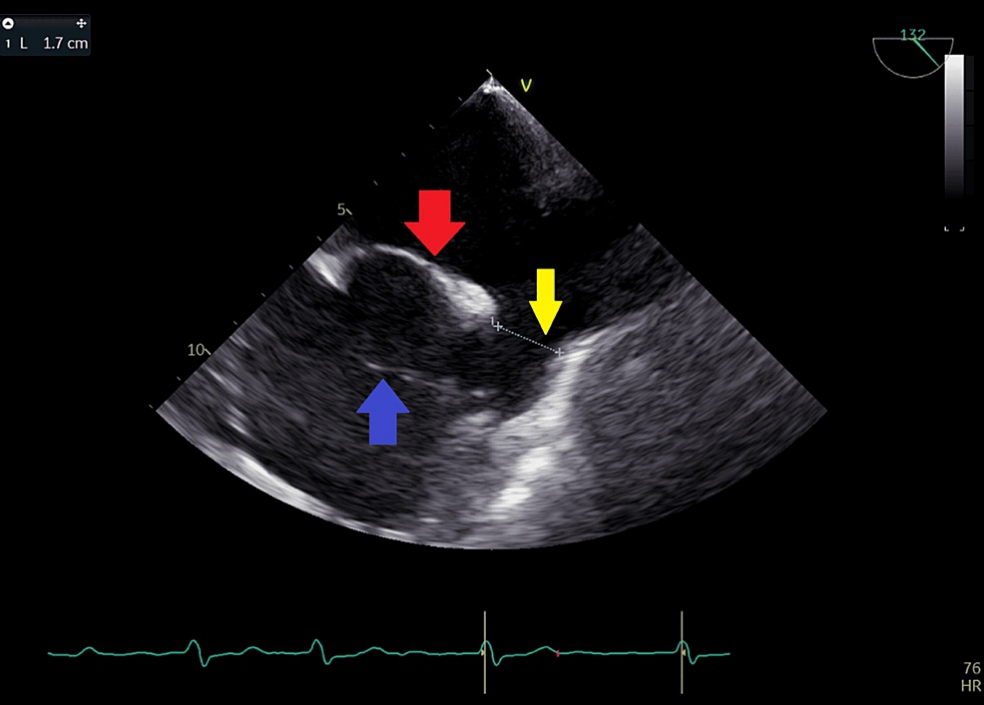
Transesophageal echocardiography at the level of left atrium and right atrium showing a 1.7 cm sinus venosus defect (yellow arrow), a cor triatriatum dexter menbrane in the right atrium (blue arrow), and an intact osteum secundum septum (red arrow) in left atrium.

**Figure 16:**
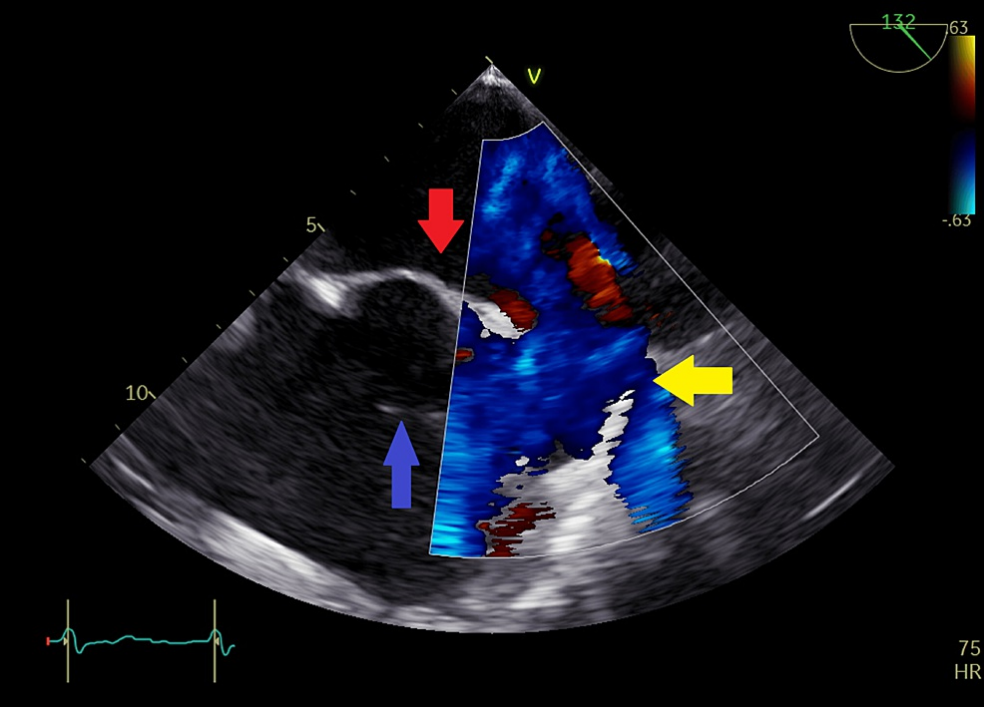
Transesophageal echocardiography at the level of left atrium and right atrium with color flow coding at the sinus venosus site showing left to right shunt across the sinus venosus atrial septal defect. Yellow arrow shows color-coded left-to-right shunt; blue arrow in right atrium shows part of the cor triatriatum dexter membrane; red arrow in left atrium shows intact osteum secundum septum

**Figure 17:**
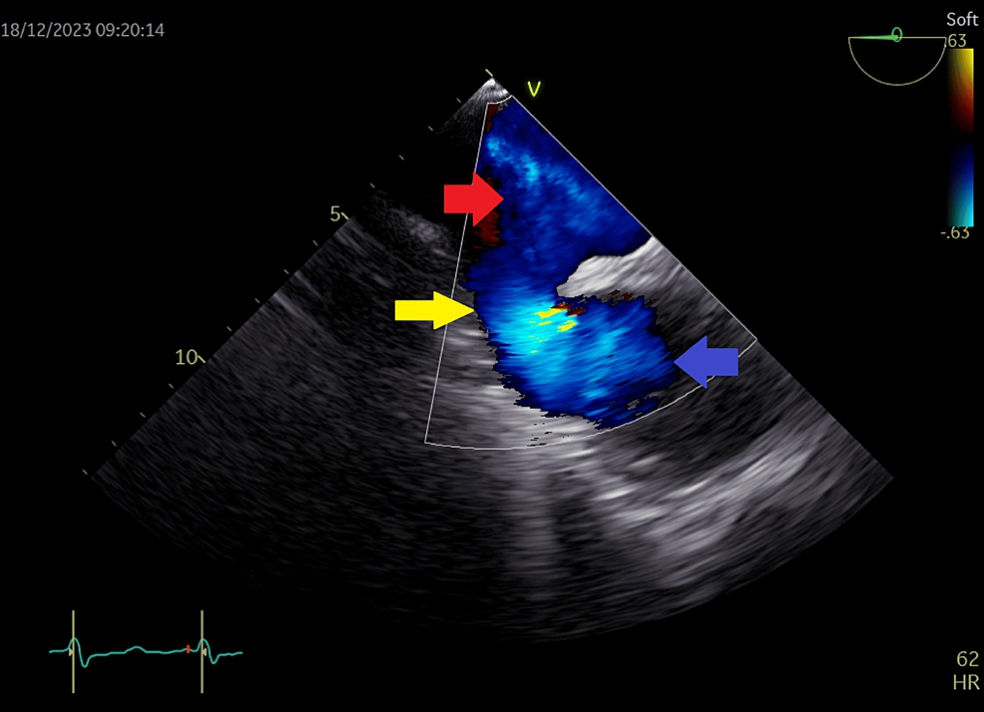
Transesophageal echocardiogram at the level of left atrium and right atrium showing color flow across the sinus venosus atrial septal defect with left to right shunt (yellow arrow). The red arrow shows flow from the left atrium to the right atrium (blue arrow).

**Figure 18:**
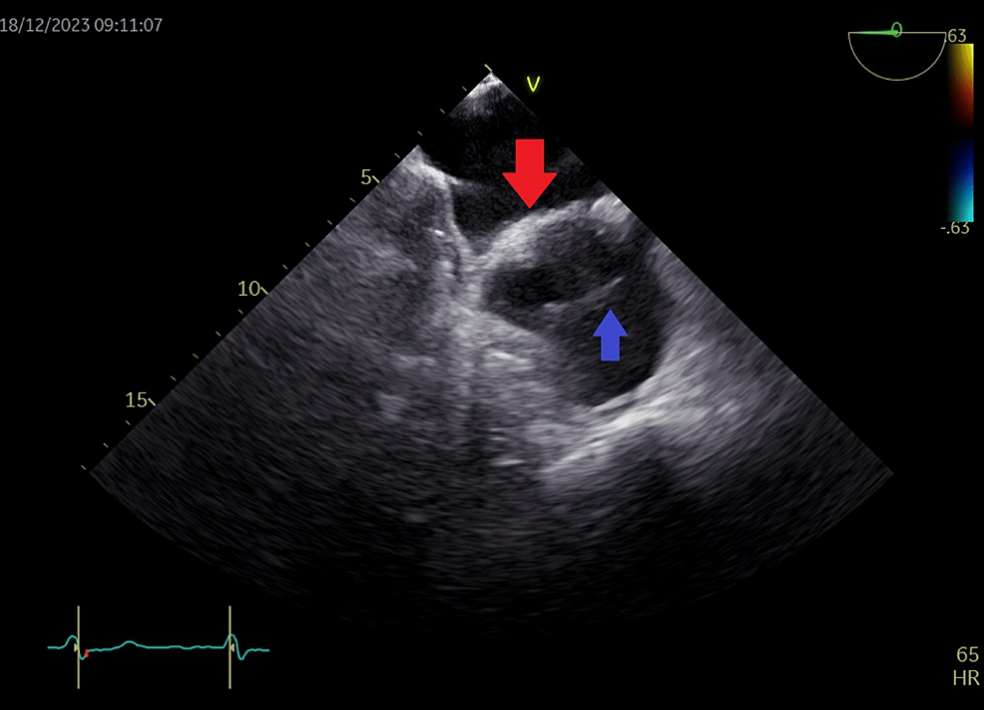
Transesophageal echocardiography view of the left atrium (red arrow) and right atrium (blue arrow) also showing the cor triatriatum dexter membrane dividing the right atrium (blue arrow).

**Figure 19:**
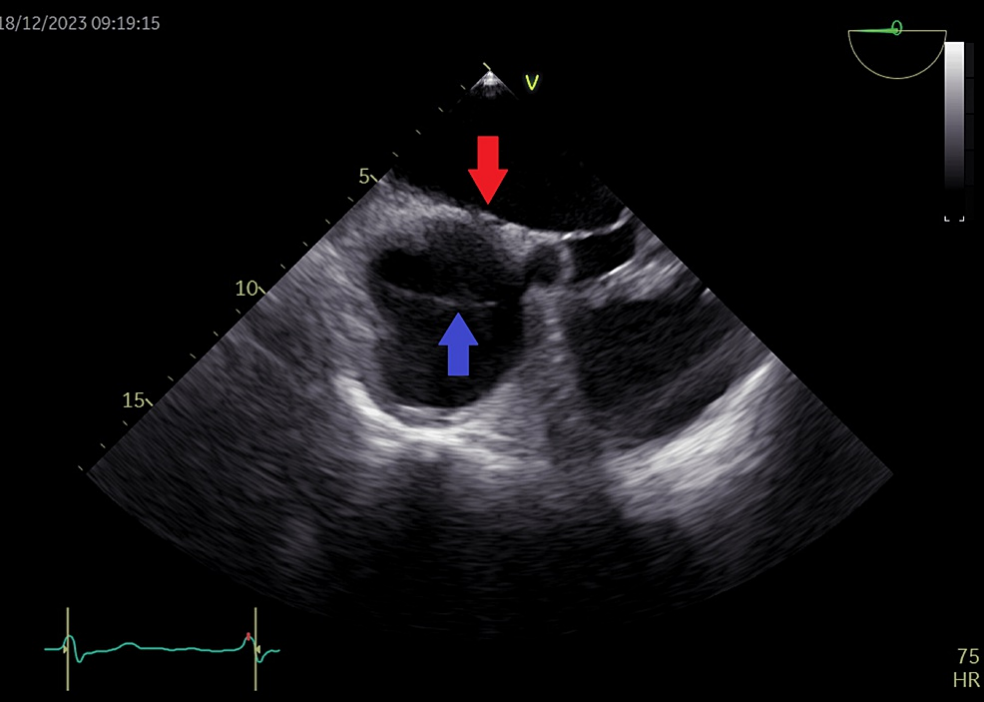
Transesophageal echocardiography view of the left atrium (red arrow) and right atrium (blue arrow) showing the cor triatriatum dexter membrane dividing the right atrium (blue arrow).

TEE was done 17 days after the presentation when the patient's condition had improved and it showed in addition to the previous findings a large 1.7 cm sinus venosus ASD (Figure 15) with a left to right shunt by color flow mapping (Figures 16, 17) and an incomplete cor triatriatum dexter membrane that was attached to the right atrial wall (Figures 15, 18, 19).

Video 1 shows the sinus venosus ASD and the incomplete cor triatriatum dexter membrane, and Video 2 shows color flow mapping across the sinus venosus ASD.

**Video 1:** Transesophageal echocardiogram of the sinus venosus atrial septal defect and the incomplete cor triatriatum dexter membrane.

**Video 2:** Color flow mapping across the sinus venosus atrial septal defect with left-to-right shunt.

All four pulmonary veins were identified going into the left atrium by TEE; it was our high suspicion of the presence of a left to right shunt at the atrial level due to an elevated Qp/Qs at 3.7 that made us do further investigation using TEE, which allowed us to detect the sinus venosus ASD and the incomplete cor triatriatum membrane. The presence of the incomplete cor triatriatum membrane was an incidental finding but it makes the percutaneous treatment of both the sinus venosus ASD and tricuspid regurgitation very difficult, so the patient was advised surgical repair and she was referred for a tertiary care center with onsite cardiac surgery capability for further testing before surgery, including cardiac CTA, cardiac MRI, coronary angiography, and right heart catheterization with invasive pulmonary artery pressure measurement looking for reversibility of pulmonary hypertension before surgical closure of sinus venosus ASD and tricuspid valve repair.

## Discussion

ASD is a well-tolerated congenital heart disease defect, frequently diagnosed later in life if not investigated by echocardiography when a cardiac murmur is present during routine physical exam in infancy or childhood [1,2]. TTE is the gold standard for the diagnosis of ASD, especially for infants and children [1].

In all patients presenting with dilated right cardiac chambers on 2D TTE, there should be a thorough investigation looking at the possibility of left-to-right shunt at the atrial level; the presence of Qp/Qs>1.5 and elevated tricuspid E velocity should orient to a left-to-right shunt at atrial level [1,2]. TEE, cardiac CTA, and cardiac MRI will help visualize the defect and associated PAVPR [5,6].

Sinus venosus ASD is difficult to diagnose by TTE [1,2], and TEE is helpful in this diagnosis. It also helps define the presence of PAVPR in the right atrium [3-5]. Cardiac CTA and cardiac MRI are gold-standard complementary imaging techniques that should be used especially before percutaneous closure of ASD for the diagnosis of associated anomalies; for example, PAVPR to the RA, SVC, or IVC. While cardiac CTA can also make accurate measurements of the ASD size and the distance from the defect to different cardiac structures (valves, aorta), cardiac MRI allows accurate calculation of the left-to-right shunt (Qp/Qs) [5,6]; these imaging techniques can also make the diagnosis of cor triatriatum dexter or sinister [8,9]. 

The interatrial septum serves as the anatomical barrier dividing the primary atrium into distinct right and left atrium. Around the fifth week of gestation, the development of the septum primum commences as it extends toward the endocardial cushions [13]. The diminishing space between the endocardial cushions and the septum primum is identified as the ostium primum. Before the fusion of the septum primum with the endocardial cushions, small perforations emerge and merge in the cephalic portion of the septum primum, forming the ostium secundum. Simultaneously, on the right side of the septum primum, the septum secundum begins to form through an invagination of the atrial wall. The growth of the septum secundum ceases by the end of the seventh week of gestation, resulting in a posterior and inferior gap known as the fossa ovalis. The lower part of the septum primum persists into adulthood as the flap valve; anomalies such as excessive apoptosis in the cephalic portion of the septum primum or incomplete development of the septum secundum can lead to an ostium secundum ASD. During birth, a decrease in right heart pressures compared to the left due to lung expansion causes the flap valve to displace against the septum secundum; in two-thirds of the population, the flap valve eventually fuses with the septum secundum, resulting in separation of the atria, the absence of fusion between the flap valve and septum secundum gives rise to a patent foramen oval (PFO) [14].

ASD types

A morphologic classification includes anomalies of the interatrial septum that establish direct communication between the right and left atria. This category includes the ostium primum ASDs, the endocardial cushion defects, and the ostium secundum ASDs. Furthermore, another classification involves defects that, while not directly involving the interatrial septum, exhibit physiological behaviors similar to traditional interatrial septal defects. This category includes sinus venosus ASDs and unroofed coronary sinus [13,14]. Careful consideration of their respective locations allows for the distinction of the diverse types of ASDs [13].

Ostium Primum ASDs

Ostium Primum ASD constitutes the mildest form among endocardial cushion defects, accounts for approximately 2-3% of ASD cases, and frequently coexists with Down syndrome. In embryonic development, the endocardial cushions contribute to the formation of the mitral and tricuspid valves, the adjacent atrial septum to the atrioventricular valves, and the ventricular septum's inlet portion. Isolated defects arising from the failure of fusion between the free edge of the septum primum and the endocardial cushion are termed "ostium primum ASDs." If such defects coincide with abnormal development of the atrioventricular valves or ventricular septum, they are categorized as endocardial cushion defects [13,14].

Ostium Secundum ASDs

Comprising 80-90% of ASDs, ostium secundum ASDs are observable defects in the fossa ovalis region. Ostium secundum ASDs present a direct communication while a PFO is a tunnel between the two atria. Distinguishing between an ostium secundum ASD and a PFO is feasible with TEE, cardiac CTA, and cardiac MRI. Normal physiological circumstances involve higher left atrial pressure compared to right atrial pressure, pressing the flap valve against the septum secundum, and narrowing the PFO tunnel. Physiologically, left-to-right PFO flow may become bidirectional under certain conditions like the Valsalva maneuver. Ostium secundum ASDs are typically larger than PFO defects, leading to enlargement of the right-sided chambers [13,14].

Sinus Venosus ASDs

Constituting approximately 2-10% of all ASDs, sinus venosus defects exhibit controversial embryological origins. However, they are generally attributed to the lack of septation between the pulmonary veins and the SVC or right atrium. Two subtypes, superior and inferior sinus venosus ASDs, involve communications between the right atrium and SVC or IVC junction and the left atrium. The superior type is often associated with PAPVR from the right upper lobe into the SVC, while the inferior type may involve PAPVR from the right lower lobe pulmonary vein into the intra-pericardial segment of the IVC or right atrium, This anomaly is sometimes associated with a persistent left SVC [13,14]. 

Unroofed Coronary Sinus

The least common type of ASD, unroofed coronary sinus results from the absence of septation between the inferior left atrium and the coronary sinus roof, facilitating communication between the left and right atria. This anomaly is frequently associated with a persistent left SVC [15].

There are familial forms of ASDs (autosomal dominant inheritance), especially when there are associated conduction abnormalities on electrocardiogram like atrioventricular (AV) block [16].

Cor triatriatum dexter membrane and sinus venosus ASD

The cor triatriatum dexter membrane and the sinus venosus ASD have different embryogenic origins [14,17]. Cor triatriatum represents an exceptionally rare congenital cardiac anomaly, accounting for a reported incidence of 0.1% among all congenital heart defects. This anomaly is categorized into two types: (i) dexter, involving the division of the right atrium, and (ii) sinister, involving the division of the left atrium. In cor triatriatum dexter, the right atrium undergoes division into two chambers due to the persistence of exaggerated fetal eustachian and thebesian valves. This occurrence stems from incomplete regression of the right valve of the right horn of the embryologic sinus venosus, leading to the formation of an incomplete septum across the lower part of the right atrium. Conversely, in cor triatriatum sinister, the left atrium is divided into two chambers by a fibromuscular septum. This results in the pulmonary veins entering a posterior-superior chamber, which is distinct from the anterior-inferior distal chamber containing the mitral valve; the controversy surrounding the origin of cor triatriatum involves two theories: the mal-incorporation theory and the entrapment theory [17]. The mere presence of the incomplete cor triatriatum dexter membrane (although asymptomatic) makes the percutaneous closure of sinus venosus ASD and tricuspid valve repair very difficult [8,9].

Our patient was misdiagnosed initially in another institution as having pulmonary disease, Cor pulmonale, and pulmonary hypertension secondary to lung disease. Using simple 2D TTE and hemodynamic measurements by Doppler, we could reorient the diagnosis toward a left-to-right shunt at the atrial level. The presence of dilatation of the right-sided chambers, the elevated E tricuspid velocity, and the Qp/Qs of 3.7 all suggested the presence of left to right shunt at the atrial level so a TEE was done that visualized the sinus venosus ASD and confirmed the presence of incomplete cor triatriatum dexter membrane and the presence of severe tricuspid regurgitation; also all four pulmonary veins were visualized going to the left atrium.

The presence of an incomplete cor triatriatum dexter membrane, despite being an innocent bystander, made the percutaneous treatment for this patient very challenging: closure of the sinus venosus ASD and repair of the tricuspid valve regurgitation [9]. Some have tried dilatation of the cor triatriatum dexter membrane during closure of ASD [8]; others have done percutaneous edge-to-edge tricuspid valve repair in a patient with cor triatriatum dexter [12], so our patient was advised surgery despite the fact that she was at increased risk due to her previous head gunshot wound injury and residual neurologic deficit. The patient was referred to a facility where onsite cardiac surgery was available, and she would undergo further investigation including cardiac CTA, cardiac MRI, and further testing regarding her pulmonary hypertension (looking at reversibility of pulmonary hypertension) before surgery. During surgery, the defects associated with the sinus venosis ASD can be identified and treated even if they were not diagnosed preoperatively [18];

We know from our literature review that this is a rare case in which a sinus venosus ASD and an incomplete cor triatriatum dexter membrane are present in the same patient. We insisted on performing the TTE that showed dilated right cardiac chambers with an elevated Qp/Qs at 3.7, which prompted the diagnosis of left-to-right shunt at the atrial level. This was followed by documentation of the sinus venosus ASD and incomplete cor triatriatum dexter membrane using TEE. Of course, more robust cardiac imaging technology like cardiac CTA and cardiac MRI would make the diagnosis of this entity very easily, but we have to insist on performing hemodynamic measurements of pulmonary and systemic flow looking for a left-to-right shunt (Qp/Qs > 1.5) in every patient with dilated right cardiac chambers using the simple 2D TTE and Doppler.

## Conclusions

The presence of dilated right-sided cardiac chambers with evidence of pulmonary to systemic flow ratio (Qp/Qs), calculated by Doppler, of more than 1.5 should prompt the diagnosis of left-to-right shunt at the atrial level associated or not with PAPVR. If pulmonary venous return is normal like in our case a percutaneous treatment could be suggested for closure of sinus venosis ASD and tricuspid valve repair. The general condition of our patient makes her surgical risk above average. The presence of an incomplete cor triatriatum dexter membrane with sinus venosus ASD in this patient is a very rare encounter and makes the percutaneous treatment very difficult, so the patient was advised a surgical correction of her cardiac defects.
